# Using Rhizosphere Phosphate Solubilizing Bacteria to Improve Barley (*Hordeum vulgare*) Plant Productivity

**DOI:** 10.3390/microorganisms9081619

**Published:** 2021-07-29

**Authors:** Ana Ibáñez, Alba Diez-Galán, Rebeca Cobos, Carla Calvo-Peña, Carlos Barreiro, Jesús Medina-Turienzo, Mario Sánchez-García, Juan José R. Coque

**Affiliations:** 1 Instituto de Investigación de la Viña y el Vino, Escuela de Ingeniería Agraria, Universidad de León, 24009 León, Spain; aibas@unileon.es (A.I.); aldig@unileon.es (A.D.-G.); rebeca.cobos@unileon.es (R.C.); ccalp@unileon.es (C.C.-P.); jmedt@unileon.es (J.M.-T.); msanchez@articai.es (M.S.-G.); 2 INBIOTEC (Instituto de Biotecnología de León), Avda. Real 1—Parque Científico de León, 24006 León, Spain; c.barreiro@unileon.es; 3 Área de Bioquímica, Departamento de Biología Molecular, Universidad de León, 24071 León, Spain

**Keywords:** phosphate solubilizing bacteria, rhizosphere, barley, crop productivity

## Abstract

On average less than 1% of the total phosphorous present in soils is available to plants, making phosphorous one of the most limiting macronutrients for crop productivity worldwide. The aim of this work was to isolate and select phosphate solubilizing bacteria (PSB) from the barley rhizosphere, which has other growth promoting traits and can increase crop productivity. A total of 104 different bacterial isolates were extracted from the barley plant rhizosphere. In this case, 64 strains were able to solubilize phosphate in agar plates. The 24 strains exhibiting the highest solubilizing index belonged to 16 different species, of which 7 isolates were discarded since they were identified as putative phytopathogens. The remaining nine strains were tested for their ability to solubilize phosphate in liquid medium and in pot trials performed in a greenhouse. Several of the isolated strains (*Advenella mimigardefordensis*, *Bacillus cereus*, *Bacillus megaterium* and *Burkholderia fungorum*) were able to significantly improve levels of assimilated phosphate, dry weight of ears and total starch accumulated on ears compared to non-inoculated plants. Since these strains were able to increase the growth and productivity of barley crops, they could be potentially used as microbial inoculants (biofertilizers).

## 1. Introduction

After nitrogen, phosphorus (P) is the second most important plant growth-limiting mineral nutrient [1]. It plays an important role in vegetal metabolic processes such as photosynthesis, respiration, macromolecular biosynthesis and disease resistance. Despite being abundantly present in soils, both in organic and inorganic forms, most P is not available for efficient root uptake because it occurs mostly in insoluble forms. In fact, less than 1% of total P in soil is considered available to plants [2]. Accordingly, P is a major limiting factor for plant growth and crop productivity [1].

It is important to note that P is a limited, non-renewable natural resource [1,2]. Indeed, P-enriched fertilizers are mainly derived from phosphate rock, which is mined and processed into valuable P-containing products. Currently, the main phosphorus reserves are located in China, Russia, USA and Morocco, while South Africa exports the largest quantity [3]. At the current rate of consumption, some authors suggest that phosphate rock reserves could be depleted over the next two centuries (more alarmist ones claim it could even be in the next 50 years) [4].

At present, the world’s population is growing quickly and accordingly crop production needs to be increased. So far, this has been achieved by intensifying the input of chemical fertilizers and pesticides. Currently, large amounts of soluble P are applied to soils as fertilizers to satisfy crop nutritional requirements. Unfortunately, most soils have a high P fixing capacity, which, together with the low P use efficiency (around 10–15%) of most crops, leads to a continued accumulation of P in soils [2]. This accumulated P is known as “legacy P”. For this reason, the amounts of P added to soil are much higher than crops actually require, making this process economically more expensive and ecologically much more damaging. It has even been argued that the phosphorus accumulated in agricultural soils would be enough to sustain maximum crop yields worldwide for about 100 years if it were completely available [5,6]. Thus, this process is unsustainable in the long term. Furthermore, P accumulation generally ends in water contamination (rivers, aquifers or even seas), in a process known as water eutrophication, and in the acidification of agricultural soils [2,5,7,8].

Therefore, the increase in global agricultural crop needs is pushing the concepts of sustainability and mitigation of environmental impacts to the forefront, and microbial inoculants-mostly denominated as biofertilizers-are gaining prominence in the agricultural framework. In contrast with chemical fertilizers, biofertilizers are usually based on plant growth-promoting rhizobacteria (PGPR) which enhance crop productivity and soil fertility without exerting any toxic effect on the environment. PGPR contribute in different ways to improving crop growth and health. They have been associated with the nitrogen fixation processes, mineral solubilization (Zn, Fe, P), improved resistance to biotic and abiotic stresses and increased fungal resistance [9,10,11] among others.

In this context, phosphate-solubilizing bacteria (PSB) have emerged in recent years as a class of PGPR that help and improve phosphorus assimilation by crops [1], and accordingly they could become an important tool for crop system sustainability, particularly as an eco-friendly strategy for increasing the bioavailability of soil P for plants. In this manuscript we describe the isolation of several PSB obtained from the barley plant rhizosphere. The best insoluble P solubilizing strains were characterized for plant growth promoting traits and tested in pot experiments to analyze their impact on P assimilation and barley plant productivity. The final objective of this work was to characterize in depth some of the isolated PSBs in order to select, based on a multi-trait analysis, the best strains for the possible future development of a biofertilizer.

## 2. Materials and Methods

### 2.1. Isolation of Bacteria from Rhizosphere of Barley Plants

Bacteria were specifically isolated from the ectorhizosphere of barley plants (Cometa cultivar) in the phenological state of maturity that had been cultivated in a field located in León (Spain) (5°35′23.84″ W and 42°34′58.356″ N), on a sandy loam-textured soil with a 4.53% organic matter content and a 7.65 pH. Five plants were gentle removing from the soil and rhizosphere soil adhered to roots was collected by introducing root fragments in a 50 mL sterile conical tube and extensive mechanical vortexing. After soil recovery tenfold serial dilutions were made in sterile water and 0.1 mL aliquots of each dilution were spread onto Nutrient Agar (Condalab, Torrejón de Ardoz, Spain), starch-casein agar [12] and Plate Count Agar (Condalab, Torrejón de Ardoz, Spain), supplemented with natamycin (200 μg/mL) to avoid fungal growth. Plates were incubated at 25 °C up to 12 days. Bacteria representative of different macro and microscopic morphological types were selected at random and conserved in Nutrient Agar plates at 4 °C until use.

### 2.2. Analysis of Phosphate Solubilization in Solid and Liquid Media

All the strains were checked for phosphate solubilization on solid media by streaked onto NBRIP (National Botanical Research Institute’s Phosphate) agar [13]. Colonies surrounded by clarification halos (halozones) were selected to analyze their solubilization index (SI) according to the protocol described by Mardad et al. [14]. Briefly, positive strains were grown on NBRIP solid medium plates, that were incubated at 30 °C for 15 days. SI was determined by measuring at 4 days of incubation the halo and the colony diameter, according the next formula: SI = (colony diameter + halozone diameter)/(colony diameter). Solubilization assays were carried out by quadruplicate. Those strains with the higher SI values were checked for phosphate solubilization in NBRIP liquid medium, using tri-calcium phosphate as sole P source. Erlenmeyer flasks (500 mL) containing 100 mL of NBRIP liquid medium were inoculated at a final concentration of 5 × 10^4^ cfu/mL with each bacterial strain tested. Assays were carried out at 30 °C and 150 rpm for 20 days. Bacterial cells and insoluble phosphate were removed by centrifugation at 14,000 rpm for 10 min. pH of the cultures were determined by pH-meter. Once the cultures were completed, samples of 100 µL of the diluted supernatant was mixed with 800 µL of 0.045% (*w*/*v*) malachite green and 100 µL of 34% (*w*/*v*) sodium citrate. Samples were incubated at 25 °C for 30 min in the dark. The absorbance of the mix samples was measured at 660 nm, and quantity of soluble phosphorous was determined by comparison with a standard curve using K_2_HPO_4_ [15]. All experiments were carried out by triplicate.

### 2.3. Identification of PSB Strains

Those bacteria with a SI above average were identified by 16S rRNA sequencing. Briefly, genomic DNA extraction was performed as described by Hopwood et al. [16]. Amplification of 16S rRNA genes was carried out using the oligonucleotides 27F and 1492R [17]. Isolates were identified by comparison with the corresponding sequences of type strains found on Ez Taxon-e database [18] (http://www.ezbiocloud.net/eztaxon/identify. Data accessed on 20 January 2017). Sequence alignment as well as phylogenetic trees were carried out using the MEGA 6 software [19].

### 2.4. Evaluation of In Vitro Antifungal Activity

The antifungal activity of the selected isolates was tested by dual culture technique as previously described [20] in 9 cm in diameter Petri dishes, against six common fungal pathogens affecting grain cereals crops such as *Alternaria* spp., *Botrytis cinerea*, *Fusarium oxysporum*, *Nigrospora oryzae, Pleospora herbarum* and *Rhizoctonia solani*. The antifungal activity was quantified by calculating the inhibition index (I index): I index (%) = [1 − (Ra − R)/(Rc − R)] × 100; where Ra is the radius of the fungal colony opposite the bacterial colony, Rc is the maximum radius of the fungal colony (farther away from the bacterial effect) and R is the radius of the agar plug containing the fungi (3.5 mm). I index values were calculated for every pathogen when the Rc value of the negative control (fungal pathogen growing in the absence of antagonist PSB) was 4.5 cm, which corresponds to a total growth in the Petri dish. Every PSB-fungal combination was tested on International Streptomyces Project 5 (ISP5) and 7 (ISP7) [21] (Shriling and Gottlieb, 1966), Potato Dextrose Agar (PDA; Condalab, Torrejón de Ardoz, Spain), Tryptic Soy Agar (TSA; Condalab, Torrejón de Ardoz, Spain) and Yeast-Extract Dextrose (YED; Condalab, Torrejón de Ardoz, Spain) agar media and the assays were carried out by triplicate.

### 2.5. Phosphatase Activity Determination

Phosphatase activities were determined as indicated by Magallon-Servin et al. [22]. Briefly, PSB were grown in 50-mL Erlenmeyer flasks containing 25 mL of TSB. Flask were inoculated with 1 mL of an overnight grown preculture, and they were incubated for 72 h at 28 °C on a rotary shaker (150 rpm). Bacterial cells were harvested and washed twice in saline by centrifugation at 14,000 rpm for 20 min. Then cells were resuspended in 2 mL Tris-buffer (pH 7.0) and sonicated (VibraCell) on ice during cycles of 2 s sonication and 15 s rest. The supernatant of sonicated cells containing phosphatases was recovered by centrifugation (14,000 rpm, 5 min at 4 °C), and used to determine enzymatic activities. Alkaline and acidic phosphatases were determined in 96-well microplates. Briefly, each plate-well received 50 μL of enzyme extract and 50 μL of substrate solution for acidic (0.05-M citrate buffer with 5.5 mM of nitrophenylphosphate at pH 4.8) or alkaline (0.05-M glycine buffer with 0.01%, (*w*/*v*) MgCl_2_ × 6H_2_O and 5.5 mM of nitrophenylphosphate at pH 10.5) phosphatase. Microplates were incubated for 10 min at 37 °C on a rotary shaker (60 rpm). Reactions were stopped by adding 200 μL of stop solution (0.5 N NaOH), giving a total reaction volume of 300 μL. The amount of *p*-nitrophenol released by the phosphatases was quantified by reading the absorbance using a Microtiter Reader at 405 nm. As a standard, 300 μL of 0.05-μmol/mL *p*-nitrophenol were added to the plate. A blank (50 μL of buffer instead of enzyme) was run in parallel to account for any possible spontaneous hydrolyzation of 4-nitrophenyl phosphate during incubation. For determining specific activities, the total soluble protein in cell extracts and supernatants was measured by the Bradford method [23] adapted to microplates.

### 2.6. Qualitative and Quantitative Estimation of Phytase Production

Isolates were tested for extracellular phytase production in Phytase Screening Medium (PSM), which contained (*w*/*v*): 2.0 D-glucose; 0.4 sodium phytate; 0.2 CaCl_2_; 0.5 NH_4_NO_3_; 0.05 KCl; 0.05 MgSO_4_ × 7H_2_O; 0.001 FeSO_4_ × 7H_2_O; 0.001 MnSO_4_ × 5H_2_O; and 2.0 agar adjusted to pH 6.0 [24]. The isolates showing clear zones on PSM were further quantified in liquid medium following the methodology described by Qvirist et al. [25]. Briefly, the isolates were grown in LB liquid medium for 7 days at 30 °C, and cells were separated from supernatant by centrifugation (8000 rpm at 4 °C for 5 min). Crude protein extract (75 µL) was incubated with 300 µL of phytate solution (1.5 mM of sodium phytate [phytic acid sodium salt hydrate] in 100 mM sodium acetate buffer, pH 5.0) for 30 min at 37 °C. The reaction was stopped by addition of 750 µL of 5% trichloroacetic acid (*w*/*v*). Color reagent (750 µL), prepared freshly by mixing 4 volumes of 1.5% ammonium molybdate (*w*/*v*) in a 5.5% (*v*/*v*) sulfuric acid solution with 1 volume of 2.7% (*w*/*v*) ferrous sulfate solution, was added to the sample solution. The production of phosphomolybdate was measured spectrophotometrically at 700 nm. Standards were prepared from KH_2_PO_4_ in bi-distilled water at phosphate concentrations from 0 to 20 mg/L.

### 2.7. Analysis of Organic Acids Production

Organic acid productions of PSB1 to PSB9 isolates was analyzed in NBRIP liquid medium. Culture filtrates were analyzed by HPLC using a Waters 996 HPLC equipped with an Alliance 2695 refractive index detector (Waters). Isocratic elution with 0.005 M H_2_SO_4_ as mobile phase was performed using a HiPlex H (300 × 7.7 mm) column (Agilent) at 50 °C and a flow rate of 0.5 mL/min. Organic acids in the samples were determined by comparing retention times and peak areas of chromatograms with the standards for Acetic, citric, formic, lactic, malic, oxalic, succinic and tartaric acids. Samples were analyzed in triplicates.

### 2.8. Determination of Miscellaneous Activities Related to PGPR Traits

Selected PSB were checked for siderophore production on CAS agar medium containing chrome azurol (CAS) and hexadecyltrimethylammonium bromide (HDTMA) as indicators [26]. The bacterial cultures were inoculated into the CAS medium and incubated at 28 ± 2 °C for 5 days. The colony with yellow-orange color halo zone was indicated positive for siderophore production. In order to achieve quantitative estimation of indole acetic acid (IAA) production cultures were grown at 30 °C and 150 rpm for 24 h. Flasks containing 100 mL of Nutrient Broth (NB) media supplemented with 1 g/L tryptophan were inoculated at 10^4^ cells/mL and incubated at 30 °C and 150 rpm for 10 days. 1.5 mL samples were taken each 24 h to determine growth spectrophotometrically and IAA production by using the Salkowski method [27]. Briefly, 1 mL of culture supernatants were mixed with 2 mL of Salkowski reagent and incubated in the dark for 25 min. DO was measured at 530 nm and it was compared with a standard curve with indoleacetic acid ranging from 0 to 100 µg/mL. Potassium solubilization was detected by using modified Aleksandrov medium [28]. Zinc solubilization was tested according to plate assays on Tris-minimal medium supplemented with zinc oxide according Sharma et al. [29]. Solubilization indexes, were estimated as the quotient between the diameter of solubilization halo zone and the diameter of the bacterial colony, multiplied by 100. HCN production in agar plates was tested as reported by Khan et al. [30]. Briefly, nutrient broth was amended with 4.4 g glycine/L and isolates were streaked on modified agar plate. A Whatmann filter paper No. 1 socked in 2% sodium carbonate in 0.5% picric acid solution was placed in the top of the plate. Plates were sealed with parafilm and incubated at 28 ± 2 °C for 4 days. Development of orange to red color indicated HCN production: paper color from yellow to light brown, brown or reddish brown was recorded for weak (+), moderate (++) and strong (+++) reaction, respectively.

### 2.9. In Vitro Germination Assay of Barley Seeds

The effect on the germination rate of barley (Cometa variety) seeds of the PSB isolated was checked by following the methodology Pastor-Bueis and colleagues [31]. Briefly, seeds were surface disinfected by immersion in 70% ethanol for 1 min and 3% (*v*/*v*) sodium hypochlorite for 5 min. After that, seeds were extensively washed with sterilized milli-Q water 3 times for 5 min. Cultures of PSB were growth in NB at 30 °C and 150 rpm for 24 h. 3M Whatman and filter paper pieces were cut fitting the diameter of a 90 mm Petri dish, and sterilized by autoclave at 121 °C for 20 min. A piece of Whatman 3M paper was put on the base of each plate, and 10 sterilized seeds were place over it. Each plate was inoculated with 3 mL of the corresponding culture and covered with a filter paper. A control treatment was also performed using sterile NB liquid medium. The Petri dishes were hermetically sealed and kept in a growth chamber at 25 °C in the dark. Seeds were incubated until control showed 80% of germination. The germination index (GI), expressed as a percentage, was calculated as the product of the relative germination percentage (RGP) and relative radicle growth (RRG). RGP = (germinated seeds at treatment)/(germinated seeds at control) × 100; RRG = (radicle growth at treatment)/(radicle growth at control) × 100. All experiments were carried out by 5 replicates.

### 2.10. Greenhouse Pot Experiments

In order to check the effect of PSB on barley crop development, in vivo pot experiments were carried out in greenhouse condition by using vermiculite as substrate, mixed with tricalcium phosphate at a 25:1 ratio. Before filling the pots, the vermiculite was washed with distillated water and sterilized twice by autoclaving at 121 °C for 20 min. Experiments were conducted in 5 L pots, with 9 replicates for each treatment. Barley seeds were surface disinfected by immersing in 70% ethanol for 1 min and 3% (*v*/*v*) sodium hypochlorite for 5 min, respectively. After that, seeds were extensively washed with sterilized milli-Q water 3 times for 5 min. Next, seeds were inoculated by immersion in a culture containing 10^8^ cfu of each individual PSB tested for 5 min, and 10 seeds were sown per pot. Negative and positive controls were not inoculated. In this case, 200 mL of modified plant nutrient solution [32], without any phosphate source, was supplied weekly to each pot. Whereas positive controls were irrigated with plant nutrient solution supplemented with 0.2 g/L of KH_2_PO_4_. For evaluation of growth promotion effect of each selected PSB, height, and fresh and dry weight of spikes, root and shoot were recorded. Dry weight was determined by placing the samples separately in an oven at 60 °C for 72 h. Assimilated phosphorous was determined at root and shoot samples as described by Watanabe and Olsen [33]. Samples were also analyzed based on FAMIC by ICP-OES 7200 (Thermo Fisher Scientific) and software Qtegra Version 2.7. Briefly, samples were displaced and aerial parts and roots were separated. Next, 0.5 g of grinded samples were weight and carefully mixed with 8 mL of ternary acid mixture (sulfuric–nitric–perchloric 1:5:2). Then, samples were digested at 400 °C for 30 min in a Bloc digest 12 (Selecta). Bidistilled water was used to bring the total reaction volume to 50 mL. Decant the samples O/N at 4 °C. Vanadate-molybdate reagent was freshly prepared by mixing 400 mL of 0.05 M ammonium heptamolybdate with 500 mL of 0.02 M ammonium metavanadate in 32.5% HNO3 and 100 mL of bidistilled water. Six mL of the reagent were mixed with 5 mL of the sample and make up to 50 mL. Soluble phosphorus was spectrophotometrically measured at 430 nm. Phosphoric acid (P_2_O_5_) was used as standard from 0 to 1% (*w*/*v*). For the starch content determination of barley grains, they were grinded by using a Retsch ball mill MM200 (Fisher Scientific). Starch content was measured following Chow and Landhäusser protocol [34]. Briefly, 50 mg of grinded sample were mixed with 5 mL 80% ethanol to remove any soluble sugar and incubated at 95 °C for 10 min. Samples were centrifuged at 2500 rpm for 5 min and supernatants were discarded (repeat twice). Pellet was dried at 50 °C for 3–4 h. In this case, 30 mg were taken and 2 mL of 0.2N KOH was added with intensive Vortex treatment in order to achieve the solubilization of starch. Samples were incubated 10 min at RT and 1200 rpm at a thermomixer, and then boiled for 30 min to complete the solubilization process. Finally, they were centrifuged 10 min at 14,000 rpm to remove any insoluble material. Supernatants were conserved at 4 °C until enzymatic digestion. Enzyme digestion was carried out on 200 µL samples by adding fresh α-amilase (Sigma-Aldrich, A-3176), following manufacture’s instruction, and incubated at 50 °C for 2 h. Samples were next mixed with 50 µL of fresh amyloglucosidase (Sigma-Aldrich, A-7420), prepared in acetate buffer at a final concentration of 15 U/mL. Samples were incubated at 55 °C for 2 h and centrifuged at 2500 rpm for 10 min. Supernatants were conserved at 4 °C. Glucose concentration was measured spectrophotometrically from 200 µL samples. Samples were mixed with 2 mL of fresh glucose oxidase/peroxidase (GO/P) reagent (Sigma-Aldrich, P 7119), and 1.6 mL of *O*-dianisidine (2.5 mg/mL) (Sigma-Aldrich, D-3252). Samples were incubated in the dark at 25 °C for 45 min. Absorbance was measured at 420 nm. Final concentration was determined by comparison with a standard curve using glucose from 0 to 1 mg/mL in 50 mM acetate buffer pH 5.0. Samples were diluted when needed whit the same buffer and tests were carried out by triplicate.

### 2.11. Statistical Analysis

Plant growth (root and shoot length and dry weight), P assimilation, barley ears production and starch richness data were tested for univariate normality using the Shapiro-Wilk test. Means were subjected to univariate analysis of variance using the One-Way ANOVA (by Scheffe post-hoc test) to determine if there were significant differences between treated and untreated plants. Finally, to measure the strength of association between the variables with significant differences, a correlation analysis using Spearman’s test was carried out. All the statistical analyses were carried out by using IMB SPSS Statistics 26.

## 3. Results

### 3.1. Isolation of Phosphate Solubilizing Bacteria on Solid Media and Their Identification

A total of 104 morphologically distinct bacteria, based on easily observable macroscopic (size, color, appearance, colony shape and pigment production) and microscopic (cellular morphology, spore production and mobility) traits were isolated from barley plant rhizosphere soil. When these strains were replicated onto NBRIP solid agar plates, 64 (61.5%) were able to produce clear halozones, indicating phosphate solubilization. This value was considerably higher than the 11.6% of PSB detected in non-rhizospheric soil of the same barley crop. The SI of the rhizosphere isolates ranged from 2.03 to 3.13, with an average of 2.30. In this case, 17 of the isolates that exhibited an above average SI were selected for molecular identification (Table 1) by partial sequencing of 16S rRNA. The 17 isolates belonged to a total of 14 different species (with 2 isolates identified as *Burkholderia fungorum*, 2 isolates as *Pseudomonas fluorescens* and 2 isolates as *Pseudomonas brassicacearum*). The sequence similarities with 16S rRNA sequences deposited in databases were always higher than 99.5% (Table 1), which is the rate of sequence similarity considered ideal for identifying a bacterial isolate at the species level [35]. All the isolates belonging to potentially dangerous species (possible plant, human or animal pathogens), (*Pseudomonas aeruginosa*, *P. brassicacearum*, *P. fluorescens*, *Pseudomonas oryzihabitans* and *Pseudomonas putida*), were discarded from further studies.

### 3.2. Phosphate Solubilization in Liquid Medium

**Figure 1:**
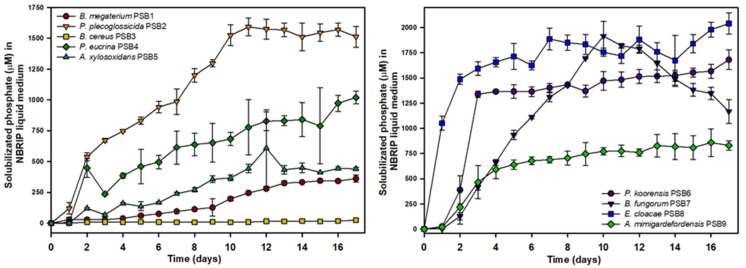
Evolution of phosphate solubilization in NBRIP liquid medium by bacterial isolates PSB1 to PSB9. Data shown (with SD) are the average of three independent experiments.

Having discarded those strains with any potential risk, the dynamics of phosphate solubilization in NBRIP liquid medium were checked in the strains listed in Table 1 as PSB1 to PSB9 (Figure 1). Under the conditions tested, the strain with the highest solubilization capacity at the end of the experiment (17 days) was *Enterobacter cloacae* PSB8 (2040.6 μM of soluble orthophosphate in culture supernatant), and *Pseudomonas koreensis* PSB6 (1680.9 µM), whereas other strains with a good solubilization capacity were *Pseudomonas plecoglossicida* PSB2 (1591.4 µM at day 11), and *B. fungorum* PSB7 (solubilization 1915.1 µM at day 10). However, under the experimental conditions, the *Bacillus cereus* PSB3 strain was not able to release a significant amount of soluble orthophosphate to the culture medium, and only a low level of soluble orthophosphate of 23.8 µM was detected at the end of the experiment. In addition, the *Bacillus megaterium* PSB1 strain exhibited a low solubilization capacity under test conditions (a maximum amount of 361.3 µM was detected at the end of experiment). The rest of the strains showed maximum amounts of soluble orthophosphate released into the culture media ranging from 609.0 µM (*Achromobacter xylosoxidans* PSB5) to 856.8 µM (*Advenella mimigardefordensis* PSB9) and 1019.5 µM (*Pantoea eucrina* PSB4).

### 3.3. Detection of Phophatase Activities Involved in P Solubilization

PSB primarily employ three different mechanisms for carrying out P solubilization: (i) liberation of phosphatase-type enzymes, including phytases; (ii) siderophore production; and (iii) organic acid production [11,36,37].

Determination of phosphatase activities involved in P solubilization was carried out. Acidic and alkaline phosphatase activities were detected in all the PSB, although these activities varied among the isolates (Table 2). *B. megaterium* PSB1 exhibited higher levels of both acidic and alkaline phosphatase, with *B. cereus* PSB3 and *P. eucrina* showing moderate levels of both activities. The rest of the strains showed relatively low extracellular phosphatase levels.

However, some of the low-phosphatase activity strains such as *A. xylosoxidans* PSB5, *A. mimigardefordensis* PSB9, *B. fungorum* PSB7 and *P. plecoglossicida* PSB2 exhibited the highest levels of phytase activity. *E. cloacae* PSB8 was the strain with the lowest capability to produce phytase and phosphatase activities.

### 3.4. Organic Acid Production by PSB

Organic acid production is one of the main mechanisms responsible for P solubilization by PSB. The production pattern of organic acids was very similar in all the PSB tested, except for *P. eucrina* PSB4, where no organic acid production was detected in NBRI liquid medium (Supplementary Material: Table S1). In this medium all the strains produced formic acid during the first 48h of fermentation at very similar levels (ranging from 0.500 mg/L to 0.408 mg/L in *B. fungorum* PSB7 and *P. plecoglossicida* PSB2, respectively). Citric acid production was detected after 72h of fermentation in *P. plecoglossicida* PSB2, *P. koreensis* PSB6, *B. fungorum* PSB7, *E. cloacae* PSB8 and *A. mimigardefordensis* PSB9. However, residual levels of tartaric acid were only detected at 48h of fermentation in PSB2 strain.

With the exception of *A. mimigardefordensis* PSB9, all the isolates tested were able to produce IAA at different levels, and *E. cloacae* PSB8 showed the highest production of this acid which is also considered to be a phytohormone (Table 3).

### 3.5. Miscellaneous PGPR Traits of PSBs

PGPR traits tested in vitro are generally associated with the ability of beneficial bacteria to promote plant growth and health. Siderophore production (involved in both iron uptake and P solubilization) was only detected in *B. megaterium* PSB1 and *B. fungorum* PSB7, whereas the only HCN producer was this last strain (Table 3). As indicated in the previous section, all the isolates tested, with the exception of *A. mimigardefordensis* PSB9, were able to produce different levels of IAA phytohormone. Three isolates (PSB2, PSB6 and PSB8) exhibited some degree of Zn solubilization efficiency, whereas the capability to solubilize K was observed at different rates in isolates PSB2, PSB6, PSB7, PSB8 and PSB9 (Table 3).

### 3.6. In Vitro Antifungal Activity

**Figure 2:**
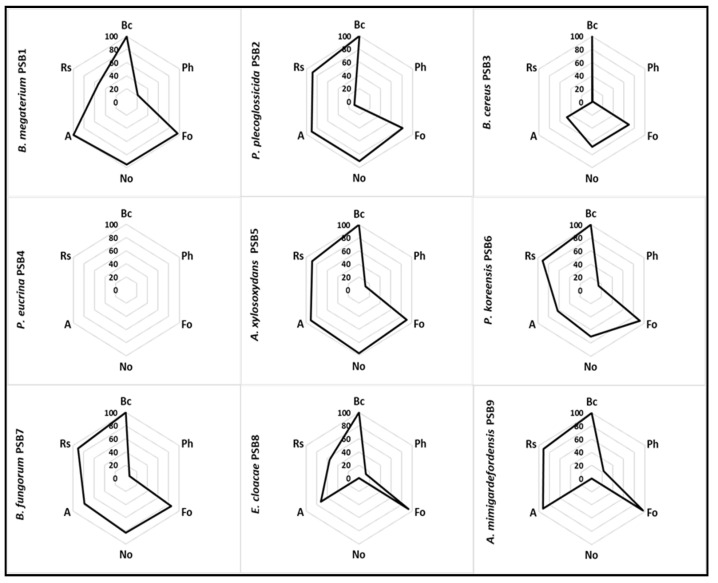
Antifungal activity of isolates PSB1 to PSB9 as determined by an in vitro bioassay (dual culture technique) on ISP5 medium. Average Inhibition index (I index) from three independent experiments are shown on a 0–100 scale. Bioassays were carried out against several phytopathogenic fungi: *B. cinerea* (Bc); *P. herbarum* (Ph); *F. oxysporum* (Fo); *N. oryzae* (No); *Alternaria* sp. (A); *R. solani* (Rs). Data shown were measured for each pathogen at different times when the Rc value of the negative control (pathogen growth in absence of antagonist PSB isolate) was 4.5 cm.

The antifungal activity of PSB1 to PSB9 isolates was checked against some of the most important crop fungal pathogens such as *Alternaria* sp., *Botrytis cinerea*, *Fusarium oxysporum*, *Pleospora herbarum*, *Nigrospora oryzae* and *Rhizoctonia solani* using a bioassay-based in vitro screening. Bioassays were carried out in several agar media, but ISP5 and ISP7 agar media were the only ones which supported the growth of all the fungal and bacterial strains used in the bioassay. The best results (shown in Figure 2) were obtained using ISP5 medium, although differences in the I index for the same pathogen were detected depending on the culture medium used (Supplementary Information Figure S1 shows the antifungal activity detected on ISP7 medium). Thus *P. eucrina* did not show any antifungal activity against any of the fungi tested on ISP5 medium (Figure 2), whereas in ISP7 medium a low antifungal activity was detected against *B. cinerea* and *P. herbarum* (Figure S1). Some of the isolates exhibited interesting antifungal properties against several of the pathogens. Thus, *B. megaterium* PSB1 exhibited total growth inhibition of *B. cinerea* and *Alternaria* sp., and good antifungal activity against *F. oxysporum* (I index 96.5) and *N. oryzae* (94.9%). In contrast, *B. cereus* PSB3 did not show any remarkable antifungal activity against either of the pathogens tested in ISP5 medium, whereas the rest of the PSB strains exhibited different rates of antifungal activity against the different pathogens tested (Figure 2).

### 3.7. In Vitro Germination Assays and Greenhouse Pot Experiments

Prior to the greenhouse pot experiments, an in vitro germination assay was carried out to check the putative effect of each individual PSB strain. No significant differences were observed as compared to inoculated seeds and negative controls. The PSB strains did not stimulate seed germination, nor did they speed up or delay the process.

Next, pot experiments were performed to analyze the capability of each PSB to solubilize insoluble P and to measure this effect on their assimilation by barley plants. The average number of plants developed per pot ranged between 7.33 (*B. cereus* PSB3 treatment) and 8.67, values obtained for both negative (supplemented with insoluble phosphate), and positive (supplemented regularly with soluble phosphate by irrigation) controls, although no significant differences were observed between treatments. Similarly, no significant differences were detected either for the spike number per pot, with values ranging from 10.67 (negative control) to 11.78 (positive control) (Supplementary information; Table S2). Once the plants had completed their growth cycle, they were uprooted and dry weight and phosphate content of their root systems were determined. Again, no significant differences were observed for both parameters (Table S2).

**Figure 3:**
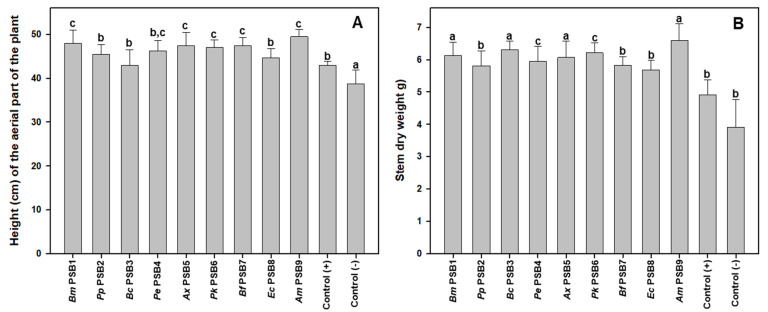
Growth data of barley plants inoculated with different PSB strains: height of the aerial part of the plant (**A**), and stem dry weight (**B**). Data shown correspond to the average of 9 replicates and each replica consisted of 10 plants sown per pot. Bars marked with the same letter are not significantly different (*p* ≥ 0.05).

However, upon visual inspection, plants revealed some clear differences in growth and development when the negative control was compared with the rest of the treatments. Indeed, mean height data for the negative control plants (Figure 3A and Figure S2) was significantly lower than for the positive control and all the batches inoculated with the different PSBs. Some of the isolates (PSB1, PSB5, PSB6, PSB7 and PSB9) were even capable of inducing greater height growth than that observed for the positive control plants (Figure 3A). Similarly, the average dry weight of the negative control plant stems was the lowest, although significant differences were only observed regarding treatments with PSB1, PSB3, PSB5 and PSB9 (Figure 3B).

**Figure 4:**
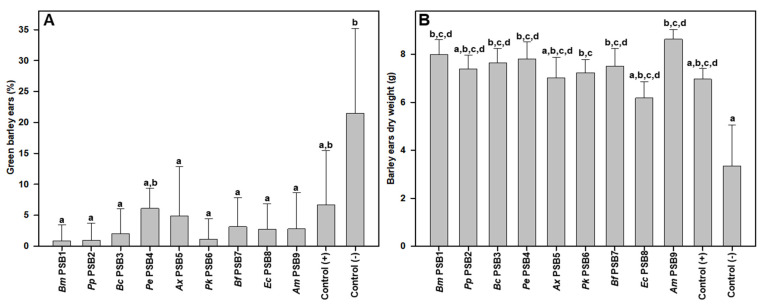
Maturation and development data of barley plant spikes inoculated with different PSB strains: percentage of immature, green barley ears at the end of the experiment (**A**) and dry weight of ears (**B**). The data shown correspond to the average of 9 replicates and each replica consisted of 10 plants sown per pot. Bars marked with the same letter are not significantly different (*p* ≥ 0.05).

Negative control plants also exhibited a deficient spike maturation level, producing a high rate of green or immature ears (Figure 4A and Figure S2). All the treatments were able to reverse this ear maturation deficiency, with significant differences for the PSB1, PSB2, PSB3, PSB5, PSB6, PSB7, PSB8 and PSB9 treatments. This result was also corroborated by the fact that the mean dry weight of the negative control spikes was the lowest observed, demonstrating significant differences with respect to PSB1, PSB3, PSB4, PSB6, PSB7 and PSB9 treatments (Figure 4B).

**Figure 5:**
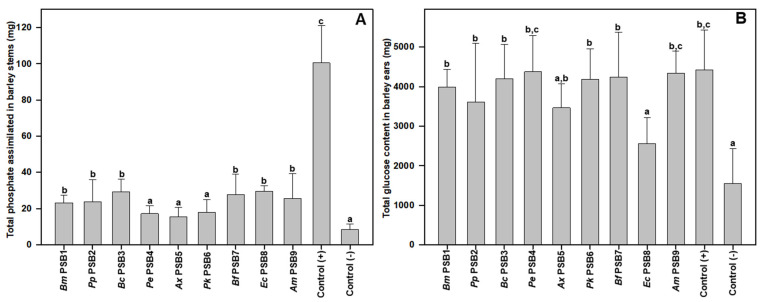
Total phosphate assimilated (**A**), and total glucose content in barley ears (**B**) at the end of the experiment. The data shown correspond to the average of 9 replicates and each replica consisted of 10 plants sown per pot. Bars marked with the same letter are not significantly different (*p* ≥ 0.05).

We also observed that the total starch/glucose content of the negative control spikes was the lowest of all the plants tested, with significant differences among all treatments, except PSB5 and PSB8 (Figure 5B). Finally, determination of assimilated phosphorus levels demonstrated that plant stems of the positive controls had the highest phosphorus content, while negative controls showed the lowest phosphorus content among all the plants measured (Figure 5A).

This deficient assimilation of phosphorus caused visually detectable symptoms in the negative control plants, whose stems frequently exhibited a purple hue indicative of such a deficiency (Figure S3). Remarkably, several of the PSB treatments (PSB1, PSB2, PSB3, PSB4, PSB6, PSB7 and PSB9) were able to significantly reverse the low level of phosphorus assimilation and accumulation observed in the stems of negative control plants, reaching phosphate assimilation levels similar to those observed in the positive control plants fertilized with soluble phosphate (Figure 5A).

Analysis of the statistical correlations between some of the variables indicated that the PSB strain variable exhibited a significant correlation (*p*-value < 0.05) with parameters such as stem height (*p*-value 0.009), stem dry weight (*p*-value 0), root dry weight (*p*-value 0.016) and spike dry weight (*p*-value 0). No significant correlation was detected between the PSB strain variable and both assimilated stem P (*p*-value 0.324), and assimilated root P (*p*-value 0.939) (Table 4).

## 4. Discussion

P-deficiency in one of the main limiting factors for crop productivity worldwide [38]. Application of soluble P-enriched chemical fertilizers satisfies the need for P in agriculture. Unfortunately, most of the soluble P added to soils is immediately mineralized and precipitated in the form of highly insoluble salts, preventing its assimilation by plants, especially at pH values higher than 5.5–6.0 [39], thus contributing to the increase of “legacy P” in soils.

Numerous bacteria have been described as phosphate solubilizers. The best known are in the *Pseudomonas*, *Burkholderia*, *Bacillus*, *Serratia* and *Enterobacter* genera, among others, and in general, all are able to increase P absorption in plants [2].

Unfortunately, not many studies have been carried out in barley (*Hordeum vulgare* L.) crops, even though barley is the fourth most widely cultivated cereal in the world, after wheat, rice and corn. Barley is mainly used as animal feed (globally, 70% of barley production is used directly or indirectly for feeding animals), whereas the rest is used for malting purposes and beer production [40].

The present report demonstrates that many different bacteria thriving in the rhizosphere environment of barley plants are able to efficiently solubilize insoluble P, including an isolate identified as *A. mimigardefordensis* PSB9, a species that, to the best of our knowledge, has not been described to date as a PSB. Up to 61.5% of the isolated bacteria exhibited some ability to solubilize P. This value is considerably higher than the 11.6% of PSB detected in bulk non-rhizospheric soil of the same barley crop analyzed. This data suggest that P solubilization could be a significant trait for bacteria thriving in rhizospheric environments and supports the hypothesis that barley, in the same way as other plants, is able to shape the root microbiome through root exudates that act as chemoattractants for a reduced group of microorganisms [41]. Our data also suggest that the rhizosphere is a strongly selective environment where bacterial diversity is significantly lower than in the bulk of the soil and also that the microbial community composition is very different [42,43].

P solubilization in liquid medium was tested for the 9 most promising strains (once strains suspected of being potentially pathogenic were discarded). Remarkably, no significant positive correlation was found between P solubilization in liquid media and the halozone produced in solid media; thus, *A. xylosoxidans* PSB5 showed the second highest levels in the solid medium assay and was the third lowest solubilizer in liquid medium. Similarly, *B. megaterium* PSB1 and *B. cereus* PSB3, which exhibited a reasonably strong solubilizing capacity in solid medium, were the two strains that showed the poorest assimilation in liquid medium. This result is concomitant with that described by other authors who state that halozone production on solid medium allowed them to recognize a PSM, but it did not provide a good quantitative estimation of P solubilizing capabilities [44,45].

All the PSB analyzed exhibited different mechanisms that were possibly responsible for P solubilization. All the strains except *P. eucrina* PSB4 were able to produce organic acids, mainly formic and citric acid, and all except *A. mimigardefordensis* PSB9 were able to produce IAA. In every strain, P solubilization in NBRIP liquid medium was accompanied by a decrease in the pH of the medium, although there was no evident direct relationship between the amount of solubilized P and the drop in pH of the medium which has also been reported in some findings [46,47]. All the strains were also able to produce phosphatases at different pH levels (both acidic and alkaline). Phosphatases induce the release of phosphorus from precipitated organic compounds in soil or fertilizers by dephosphorylating phospho-ester or phosphoanhydride bonds. This hydrolysis removes a phosphate ion, generating a molecule with a free hydroxyl group and soluble phosphate [1,2,48]. In addition, all the strains exhibited phytase activity, responsible for phytic acid degradation, which is the main way in which plants store phosphate in their tissues, pollen and seeds [24]. Together, these enzymes are involved in P release from organic matter by hydrolysis of C-O-P ester bonds, which are therefore important in P nutrition for plants [39]. In our trial, which took place in vermiculite (a mineral substrate with residual organic matter content), it could be expected that the role of phytases in P assimilation would be merely testimonial once the seed was germinated and the seedling formed. However, phytases could have a preponderant role in the mobilization of P from the phytic acid accumulated in seeds as a P reserve, which is more than sufficient to cover the developmental needs of the embryo and the incipient seedling. Therefore, we can assume that the ability of each particular strain to solubilize P would be due to a combination of different mechanisms, but that the proportion of each type of mechanism used would be different in each strain analyzed.

Given the observed P solubilization capacities of the selected strains in both solid and liquid media, we decided to analyze their possible efficacy in solubilizing P and allowing its effective incorporation into a barley crop grown in pots under greenhouse conditions. As expected, barley plants grown in a substrate with insoluble phosphate as the only source of P (negative control plants) showed visually clear symptoms of deficient P assimilation, and also a significant reduction in plant height, compared to crops grown from seeds soaked in suspensions of the different PSB tested. Not only that, but the negative control plants also showed a notorious inability to reach full ear maturation by the end of the experiment, with significant differences with respect to all treatments except the positive control and the *P. eucrina* PSB4 treatment. In addition, the dry weight of barley ears was significantly lower than that of ears treated with PSB1, PSB3, PSB4, PSB6, PSB7 and PSB9. These data showed that, to a greater or lesser extent, most PSB tested could reverse the poor growth observed in the negative control plants.

Interestingly, analysis of statistical correlations between the different parameters indicated that PSB strains had a significant impact on growth and development parameters such as stem height, stem dry weight, root dry weight and spike dry weight, thus very positively influencing crop development. However, no significant statistical correlation was detected between the amount of P assimilated in stems and any other of the parameters. Regarding P assimilated in roots, the only significant correlation detected was with the stem height parameter. These data are interesting and indicate that P uptake alone is not enough to explain the growth improvement data observed. In fact, as suggested by other researchers, the ability to solubilize P is not necessarily correlated with a higher P content in the plant, and with the ability to promote plant growth, since growth promotion can be the outcome of other mechanisms [45,49]. Accordingly, all the PSB tested exhibited, in different ranges and combinations, different traits considered typical of PGPR such as HCN, IAA and siderophore production, or solubilization of other cations such as K and Zn, that could also contribute to stimulating plant development, and given their in vitro antifungal activity, could also help control some pathogens. Therefore, a dual selection based on both plant growth-promoting traits and antifungal production should be particularly desirable in controlling some of the most important fungal pathologies affecting barley crops. The beneficial effects on barley growth observed after seed soaking with PSB may be directly due to an increase in available soluble P, or indirectly to changes in the growth rate and metabolic activities of the crop induced as a result of collateral effects triggered by other plant growth promoting traits [39].

Most of the PSB tested were able to significantly increase crop productivity in terms of ear dry weight (PSB1, PSB3, PSB4, PSB6, PSB7 and PSB9), and starch (glucose) content (PSB1, PSB2, PSB3, PSB4, PSB6, PSB7 and PSB9) as compared to negative controls, reaching similar productivity levels to those observed in positive control plants fertilized with soluble P. Thus, the significant increase in starch accumulation in ears ranged from 179.5% for *A. mimigardefordensis* PSB9 to 132.6% for *P. plecoglossicida* PSB2. These remarkable increases in productivity were a consequence of incorrect maturation of many of the ears in the negative control plants, which showed a very high level of green or immature ears. Such increases in productivity will be difficult to achieve in field-grown crops. In fact, Zaballa et al. [45] described a 16.8% increase in productivity of barley crops treated with the *Enterobacter ludwigii* PSB, where productivity was estimated as the average weight of 1000 barley grains. Furthermore, Sahin and colleagues [50] could observe a 7.5% yield increase in barley crops treated with the Bacillus M-13 strain PSB.

As indicated before, legacy P is a significant potential secondary P source that is currently underutilized. It is obvious that P-solubilization in fields tends to be much more difficult to demonstrate than in the laboratory, since P-solubilizing activity could be influenced by many different factors such as the soil’s chemical composition, physical properties, pH, salinity, organic matter content, other microbial populations, etc. [1]. Nevertheless, pot experiments showed that a wide variety of bacteria isolated from the barley plant rhizosphere can efficiently solubilize insoluble P and incorporate it into plant material. Some, such as *A. mimigardefordensis* PSB9 (not described before as PSB), were best able to increase barley yield. Other isolates such as *B. megaterium* PSB1 and *B. cereus* PSB3, that could also significantly increase the yield of barley plants were particularly interesting because of their capability to produce endospores, and therefore survive under difficult environmental conditions. Production of heat resistant endospores is also very attractive, since endospores can survive manufacture and storage conditions in such a way that the microorganism could be easily incorporated into the field on coated seeds [51]. It should, therefore, not be surprising that in the coming years the agricultural use of PSB will increase as they are applied on different crops as a viable alternative and cost-effective way of stimulating the bioavailability of the P accumulated in soils as “legacy P”.

## 5. Conclusions

The main conclusions of this study are as follows: 1. -Barley plant rhizosphere is an environment from which a wide variety of phosphate solubilizing bacteria can be isolated; 2. -Many of the isolated PSB also exhibited other plant growth promoting traits; 3. -PSB application to plants grown in a medium with insoluble P as the only source of P allowed its correct solubilization and assimilation; 4. -Plants grown in the presence of PSB showed a much higher productivity than non-inoculated plants; 5. -Finally, this work demonstrates that PSB use is a promising strategy for capitalizing on the currently unused legacy P present in soils.

## Figures and Tables

**Table 1:** Bacterial strains isolated from the barley plant rhizosphere and selected based on their higher-than-average phosphate solubilization index (SI) in solid BRIP medium and molecular identification by 16S rRNA sequencing.

Isolate	16S rRNA Sequence Similarity (%)	GenBank Accession No.	Solubilization Index (SI) in Solid NBRIP Medium *
*Bacillus megaterium* PSB1	100.00%	MZ229443	2.30 (0.06)
*Pseudomonas plecoglossicida* PSB2	99.90%	MZ229444	2.44 (0.14)
*Bacillus cereus* PSB3	99.91%	MZ229445	2.59 (0.03)
*Pantoea eucrina* PSB4	99.71%	MZ229446	2.62 (0.09)
*Achromobacter xylosoxidans* PSB5	99.63%	MZ229447	3.00 (0.22)
*Pseudomonas koreensis* PSB6	100.00%	MZ229448	2.67 (0.08)
*Burkholderia fungorum* PSB7	99.91%	MZ229449	3.13 (0.12)
*Enterobacter cloacae* PSB8	99.90%	MZ229450	2.62 (0.21)
*Advenella mimigardefordensis* PSB9	99.51%	MZ229451	2.50 (0.06)
*Pseudomonas brassicacearum* PSB10	100.00%	MZ229452	2.86 (0.09)
*Pseudomonas fluorescens* PSB11	99.90%	MZ229453	2.37 (0.10)
*Pseudomonas oryzihabitans* PSB12	100.00%	MZ229454	2.57 (0.02)
*Pseudomonas putida* PSB13	99.90%	MZ229455	2.81 (0.07)
*Pseudomonas aeruginosa* PSB14	99.80%	MZ229456	2.31(0.13)
*Burkholderia fungorum* PSB15	99.91%	MZ229457	2.50 (0.03)
*Pseudomonas fluorescens* PSB16	99.81%	MZ229458	2.87 (0.09)
*Pseudomonas brassicacearum* PSB17	100.00%	MZ229459	2.62 (0.06)

* Values shown are the average (standard deviation) of four independent solubilization assays.

**Table 2:** Extracellular phosphatase and phytase activities detected in the PSB isolates.

Isolate	Acidic Phosphatase * (mU/μg Protein)	Alkaline Phosphatase * (mU/μg Protein)	Phytase * (U/mL)
*B. megaterium* PSB1	80.83 (1.9)	94.10 (11.6)	67.7 (10.2)
*P. plecoglossicida* PSB2	4.70 (0.3)	2.70 (0.07)	99.2 (17.7)
*B. cereus* PSB3	29.00 (3.6)	10.70 (2.9)	47.2 (11.4)
*P. eucrina* PSB4	34.62 (1.6)	41.54 (3.4)	64.0 (12.6)
*A.* PSB5	0.91 (0.1)	1.56 (0.7)	143.0 (14.7)
*P. koreensis* PSB6	1.55 (0.2)	2.45 (0.4)	57.4 (9.4)
*B. fungorum* PSB7	2.86 (0.4)	2.88 (0.7)	110.1 (33.6)
*E. cloacae* PSB8	0.91 (0.2)	0.95 (0.3)	40.6 (5.1)
*A. mimigardefordensis* PSB9	2.72 (1.1)	2.91 (0.6)	135.6 (48.2)
*P. brassicacearum* PSB10	80.83 (1.9)	94.10 (11.6)	67.7 (10.2)
*P. fluorescens* PSB11	4.70 (0.3)	2.70 (0.07)	99.2 (17.7)
*P. oryzihabitans* PSB12	29.00 (3.6)	10.70 (2.9)	47.2 (11.4)
*P. putida* PSB13	34.62 (1.6)	41.54 (3.4)	64.0 (12.6)
*P. aeruginosa* PSB14	0.91 (0.1)	1.56 (0.7)	143.0 (14.7)
*B. fungorum* PSB15	1.55 (0.2)	2.45 (0.4)	57.4 (9.4)
*P. fluorescens* PSB16	2.86 (0.4)	2.88 (0.7)	110.1 (33.6)
*P. brassicacearum* PSB17	0.91 (0.2)	0.95 (0.3)	40.6 (5.1)

* Values shown are the average (standard deviation) of two independent experiments performed in triplicate

**Table 3:** Miscellaneous activities of the selected PSB related to PGPR traits including siderophore and HCN production, and ability to solubilize Zn and K.

Isolate	Siderophores Production	HCN Production	IAA Production (μg/mL) *	Zn Solubilization Efficiency (%) *	K Solubilization Efficiency (%) *
Bacillus megaterium PSB1	+	-	41.62 (0.33)	-	-
Pseudomonas plecoglossicida PSB2	-	-	14.63 (7.22)	143.82 (1.1)	356.25 (72.5)
Bacillus cereus PSB3	-	-	17.30 (1.95)	-	-
Pantoea eucrina PSB4	-	-	11.97 (0.01)	-	-
Achromobacter xylosoxidans PSB5	-	-	1.16 (0.17)	-	-
Pseudomonas koreensis PSB6	-	-	23.05 (1.27)	194.44 (37.1)	480.16 (53.1)
Burkholderia fungorum PSB7	+	+	24.13 (1.95)	-	317.22 (26.7)
Enterobacter cloacae PSB8	-	-	88.52 (1.44)	146.23 (25.4)	582.97 (54.0)
Advenella mimigardefordensis PSB9	-	-	-	-	221.43 (27.4)

* Values shown are the means (standard deviation) of two independent experiments performed in triplicate.

**Table 4:** Statistical correlation between some of the variables analyzed in the pot experiment. The results shown correspond to Spearman’s correlation coefficient. Those data with statistical significance (*p* value < 0.05) are highlighted in blue.

	Strain	Stem Height	Stem Dry Weight	Assimilated Stem P	Root Dry Weight	Assimilated Root P	Ears Dry Weight	Glucose in Ears
Strain	1							
Stem height	0.009	1						
Stem dry weight	0	0	1					
Assimilated stem P	0.324	0.249	0.491	1				
Root dry weight	0.016	0	0	0.394	1			
Assimilated root P	0.939	0.004	0.351	0.163	0.116	1		
Ears dry weight	0	0	0	0.610	0	0.016	1	
Glucose in ears	0.738	0.937	0.231	0.931	0.251	0.985	0.010	1

## Data Availability

Not applicable.

## Supplementary Materials

The following are available online at https://www.mdpi.com/article/10.3390/microorganisms9081619/s1: Figure S1: Antifungal activity of isolates PSB1 to PSB9 determined by an in vitro bioassay (dual culture technique) on ISP7 medium title; Figure S2: General appearance of barley plants inoculated with the *Burkholderia fungorum* PSB7 strain (left) and negative control plants (right); Figure S3: General appearance of negative control barley plants and enlarged detail of a stem. Table S1: Organic acid production by selected PSB in liquid NBRIP medium. Table S2: Different parameters of barley plants inoculated with different PSB strains in the pot experiment.
