# Supplementary material for: Using Rhizosphere Phosphate Solubilizing Bacteria to Improve Barley (Hordeum vulgare) Plant Productivity
Source: Microorganisms. 2021 Jul 29;9(8):1619. doi: 10.3390/microorganisms9081619 (PMC8401182; doi:10.3390/microorganisms9081619)
Supplement: Supplementary file 1 [file microorganisms-09-01619-s001.zip › S2 Figure-Ibañez et al 2022.pdf]

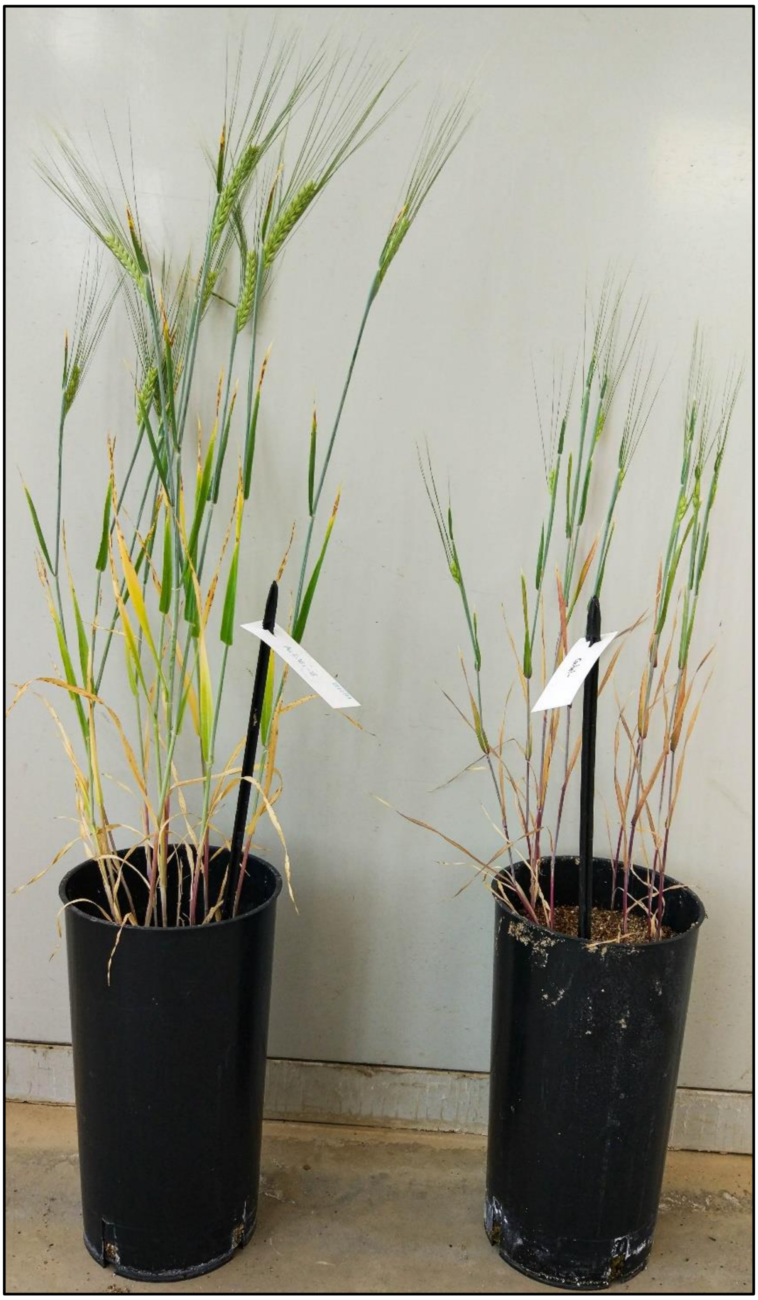

Supplementary information S2 Figure.- General appearance of barley plants inoculated with the *Burkholderia fungorum* PSB7 strain (left), and plants corresponding to the negative control (right). Note the great difference regarding the development in height of the plants and how the ears of the negative control are not able of reaching a correct maturity.
