# Supplementary material for: Using Rhizosphere Phosphate Solubilizing Bacteria to Improve Barley (Hordeum vulgare) Plant Productivity
Source: Microorganisms. 2021 Jul 29;9(8):1619. doi: 10.3390/microorganisms9081619 (PMC8401182; doi:10.3390/microorganisms9081619)
Supplement: Supplementary file 1 [file microorganisms-09-01619-s001.zip › S3 Figure-Ibañez et al 2021.pdf]

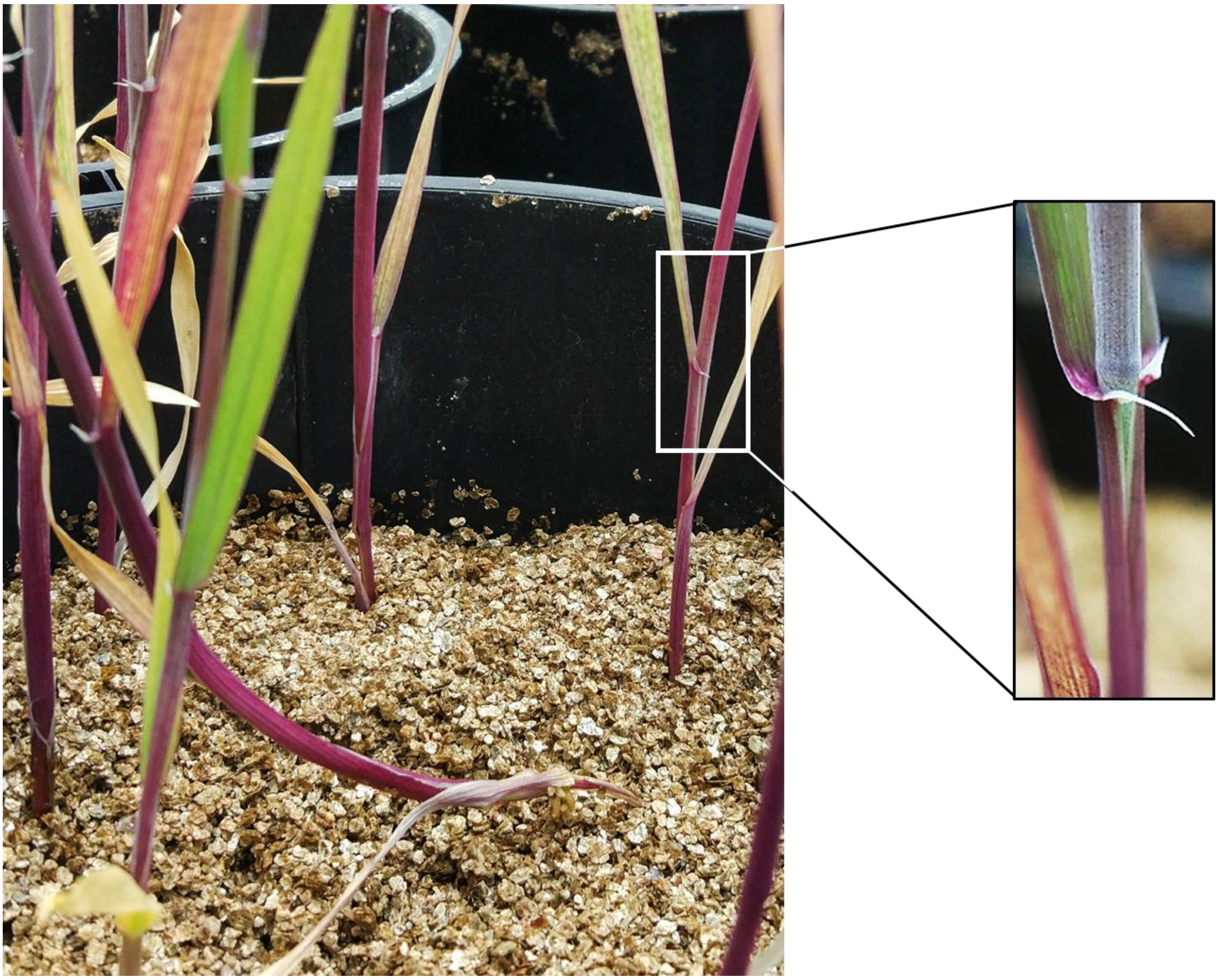

Supplementary information S3 Figure.- General appearance of barley plants corresponding to the negative control, and enlarged detail of a stem. Note the visually detectable typical symptoms of a deficient assimilation of phosphorus resulting in a purple hue of the stems.
