# Supplementary material for: Using Rhizosphere Phosphate Solubilizing Bacteria to Improve Barley (Hordeum vulgare) Plant Productivity
Source: Microorganisms. 2021 Jul 29;9(8):1619. doi: 10.3390/microorganisms9081619 (PMC8401182; doi:10.3390/microorganisms9081619)
Supplement: Supplementary file 1 [file microorganisms-09-01619-s001.zip › Table S1-Ibañez et al 2021.pdf]

Table S1. Different parameters of barley plants inoculated with different PSB strains in pot experiment

Isolate	Number or plants per pot*	Number of ears per pot*	Average dry weight per ear (g)[#§]	Average roots dry weight (g)[#§]	Total phosphate assimilated in root system (mg) [#§]
Bacillus megaterium PSB1	8.22 (0.67)	11.67 (1.22)	0.69 (0.06)[b]	1.85 (0.17)[a,b]	1.26 (0.31)[a,b]
Pseudomonas plecoglossicida PSB2	8.56 (1.01)	11.44 (1.13)	0.65 (0.09)[b]	1.86 (0.17)[a,b]	0.87 (0.24)[a,b]
Bacillus cereus PSB3	7.33 (1.12)	11.00 (0.87)	0.70 (0.06)[b]	1.99 (0.48) [a,b]	0.74 (0.24)[a,b]
Pantoea eucrina PSB4	7.89 (0.78)	11.56 (1.42)	0.68 (0.06)[b]	2.48 (0.64)[b]	1.18 (0.56)[a,b]
Achromobacter xylosoxidans PSB5	8.22 (0.87)	11.56 (1.81)	0.62 (0.12)[b]	2.44 (0.51)[b]	0.95 (0.32)[a,b]
Pseudomonas koreensis PSB6	8.33 (1.22)	11.11 (0.78)	0.65 (0.05)[b]	2.17 (0.55)[a,b]	0.70 0.23)[a,b]
Burkholderia fungorum PSB7	8.11 (1.27)	10.33 (0.87)	0.73 (0.10)[b]	2.43 (0.66)[b]	1.39 (0.76)[a,b]
Enterobacter cloacae PSB8	9.00 (1.22)	11.67 (1.66)	0.53 (0.05)[b]	1.95 (0.19)[a,b]	1.16 (0.87)[a,b]
Advenella mimigardefordensis PSB9	8.22 (1.09)	11.67 (1.00)	0.29 (0.23)[a]	2.69 (1.34)[b]	1.68 (0.65)[a,b]
Control (+)	8.67 (0.71)	11.78 (1.09)	0.59 (0.06)[b]	1.57 (0.20)[a,b]	1.56 (0.82)[b]
Control (-)	8.67 (0.87)	10.67 (1.32)	0.31 (0.14)[a]	1.17 (0.27)[a]	0.49 (0.29)[a]

* Values showed are the means (standard deviation) of two independent experiments made by triplicate
[#] Values showed are the means (standard deviation) of two independent experiments made by triplicate
§ Values marked with the same letter are not significantly different ($P \geq 0.05$)
