# Supplementary material for: Using Rhizosphere Phosphate Solubilizing Bacteria to Improve Barley (Hordeum vulgare) Plant Productivity
Source: Microorganisms. 2021 Jul 29;9(8):1619. doi: 10.3390/microorganisms9081619 (PMC8401182; doi:10.3390/microorganisms9081619)
Supplement: Supplementary file 1 [file microorganisms-09-01619-s001.zip › Table S2-Ibañez et al 2021.pdf]

Supplementary information: Table S2. Organic acid production by selected PSB in liquid NBRIP medium.

		24 h	48 h	72 h	96 h	120 h	144 h
***Bacillus megaterium* PSB1**	Citric acid						
	Formic acid	0.492*	0.468				
	Tartaric acid						
***Pseudomonas plecoglossicida* PSB2**	Citric acid			0.220	0.418	0.484	0.655
	Formic acid	0.408	0.474				
	Tartaric acid		0.04				
***Bacillus cereus* PSB3**	Citric acid						
	Formic acid	0.448	0.813				
	Tartaric acid						
***Pantoea eucrina* PSB4**	Citric acid						
	Formic acid						
	Tartaric acid						
***Achromobacter xylosoxidans* PSB5**	Citric acid						
	Formic acid	0.443	0.467				
	Tartaric acid						
***Pseudomonas koreensis* PSB6**	Citric acid			0.353	0.364	0.34	0.284
	Formic acid	0.52	0.517				
	Tartaric acid						
***Burkholderia fungorum* PSB7**	Citric acid			0.502	0.748	0.796	0.935
	Formic acid	0.500	0.513				
	Tartaric acid						
***Enterobacter cloacae* PSB8**	Citric acid			0.115	0.028	0.022	0.022
	Formic acid	0.478	0.501				
	Tartaric acid						
***Advenella mimigardefordensis* PSB9**	Citric acid			0.021	0.027	0.033	0.029
	Formic acid	0.489	0.520				
	Tartaric acid						

* Values shown are expressed in mg/L and correspond to the average of three independent experiments
