# Supplementary material for: Using Rhizosphere Phosphate Solubilizing Bacteria to Improve Barley (Hordeum vulgare) Plant Productivity
Source: Microorganisms. 2021 Jul 29;9(8):1619. doi: 10.3390/microorganisms9081619 (PMC8401182; doi:10.3390/microorganisms9081619)
Supplement: Supplementary file 1 [file microorganisms-09-01619-s001.zip › S1 Figure-Ibañez et al 2021.pdf]

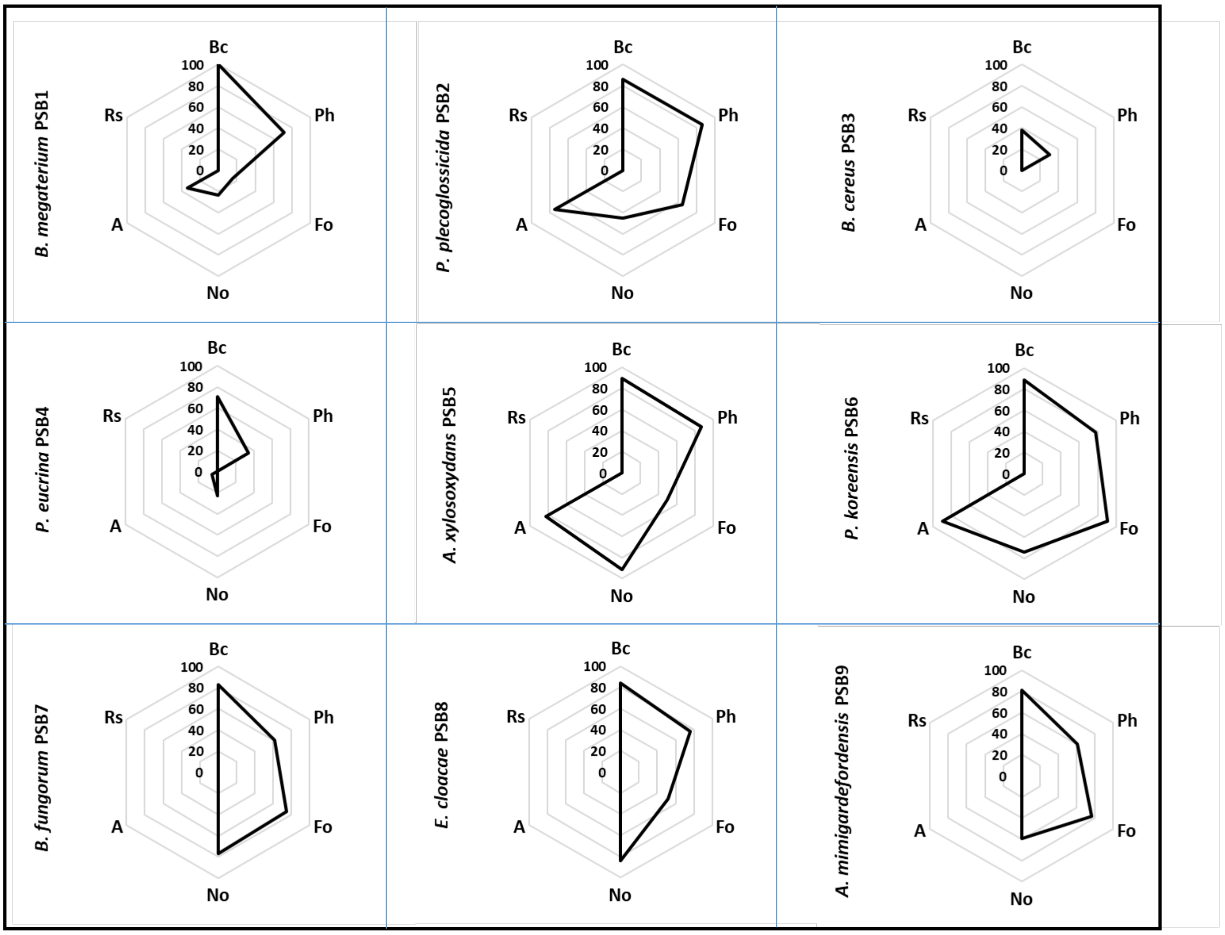

Supplementary information S1 Figure.- Antifungal activity of isolates PSB1 to PSB9 as determined by an *in vitro* bioassay (dual culture technique) on ISP7 medium. Average Inhibition index (I index) from three independent experiments are shown on a 0-100 scale. Bioassays were carried out against several phytopathogenic fungi: *B. cinerea* (Bc); *P. herbarum* (Ph); *F. oxysporum* (Fo); *N. oryzae* (No); *Alternaria* sp. (A); *R. solani* (Rs). Data shown were measured for each pathogen at different times when the Rc value of the negative control (pathogen growth in absence of antagonist PSB isolate) was 4.5 cm.
